# Pre-Treatments Involving Aqueous Ozone and UV-C Light Can Be Used in Raisin Production to Decrease the Incidence of *Aspergillus carbonarius* and Promote Drying

**DOI:** 10.3390/foods15030550

**Published:** 2026-02-04

**Authors:** Eunice Valentina Contigiani, Angela Rocío Romero Bernal, Paula Sol Pok, Analía Belén Garcia Loredo, María Bernarda Coronel, Stella Maris Alzamora, Paula Luisina Gómez

**Affiliations:** 1 Instituto de Tecnología de Alimentos y Procesos Químicos (ITAPROQ), Consejo Nacional de Investigaciones Científicas y Técnicas (CONICET), Universidad de Buenos Aires, Ciudad Universitaria, Ciudad Autónoma de Buenos Aires C1428, Argentina; eunicecontigiani@gmail.com (E.V.C.); romerobangela@gmail.com (A.R.R.B.); mbcoronel@di.fcen.uba.ar (M.B.C.); 2 Departamento de Industrias, Facultad de Ciencias Exactas y Naturales, Universidad de Buenos Aires, Ciudad Universitaria, Ciudad Autónoma de Buenos Aires C1428, Argentina; 3 Facultad de Ingeniería, Universidad de Buenos Aires, Ciudad Universitaria, Ciudad Autónoma de Buenos Aires C1063, Argentina; 4 Instituto de Tecnología de Alimentos, Instituto Nacional de Tecnología Agropecuaria (INTA), Nicolas Repetto y de los Reseros s/n, Hurlingham B1686, Buenos Aires, Argentina; 5 Facultad de Ciencias Médicas, Pontificia Universidad Católica Argentina, Av. Alicia Moreau de Justo 1500, Ciudad Autónoma de Buenos Aires C1107, Argentina; 6 Facultad de Ingeniería, Universidad Nacional de Mar del Plata, Juan B. Justo 4302, Mar del Plata B7600, Buenos Aires, Argentina

**Keywords:** grape, drying kinetics, novel pre-drying treatments, fungal contamination, epicuticular waxes, quality

## Abstract

In order to reduce fungal contamination in grapes and increase the dehydration rate for producing raisins, the development of alternative technologies that do not compromise product safety and quality is required. This study examined the impact of innovative pre-drying methods using aqueous ozone (10 min-4.1 mg O_3_ L^−1^) and UV-C light (30.3 kJ m^−2^ UV-C) on the incidence of *Aspergillus carbonarius*, as well as on air-drying kinetics and ultrastructure of epicuticular waxes in Sultanina grapes, when applied either individually or sequentially. The effect of the pre-treatments on the colour of the dehydrated grapes was also assessed. Grapes pre-treated with 30.3 kJ m^−2^ UV-C and 10 min-4.1 mg O_3_ L^−1^ + 30.3 kJ m^−2^ UV-C showed a lower incidence of *A. carbonarius* in storage at 20 ± 1 °C than those exposed to aqueous ozone (30 and 8% lower infection compared to the non-pretreated fruit at 15-day storage, respectively). Although the combined pre-treatment did not significantly improve the fungus inhibition with respect to the individual UV-C application, it allowed a higher dehydration rate during the drying process at 60 ± 1 °C. The drying time was reduced by ~31% compared to non-pretreated fruit, a result slightly lower than that achieved with the traditional chemical pre-treatment of ethyl oleate-K_2_CO_3_ (~39%). This enhancement in drying rate was partly attributed to marked alterations in the grape’s epicuticular wax layer. UV-C and the combined pre-treatment helped in reducing the browning of raisins. Therefore, the combined application of ozone and UV-C light could be an environmentally friendly alternative for both improving the microbiological quality of grapes and accelerating the drying process.

## 1. Introduction

Raisins or dried grapes are consumed worldwide and recognised for their beneficial health properties. They are a rich source of fibre and phenolic compounds, including phenolic acids, catechin, procyanidins, and quercetin, which are linked to their prebiotic effect [1]. Raisin consumption has been shown to prevent many diseases, including cancer, cardiovascular disease, and diabetes [2,3,4]. Different types of raisins are produced according to the variety of grape used. The Sultanina grape variety (also known as Thompson Seedless) remains one of the most important varieties for the production of raisins, valued for its elongated oval shape and early maturation, despite drying more slowly compared to other varieties [5].

Fungal contamination of grapes can adversely affect the quality and safety of raisins, potentially promoting mycotoxin contamination. In this regard, a primary concern in these products is the presence of the mycotoxin Ochratoxin A (OTA) [6,7]. This mycotoxin exhibits nephrotoxic, immunotoxic, genotoxic, neurotoxic, and teratogenic properties, and has been classified by the International Agency for Research on Cancer (IARC) as a possible Group 2B human carcinogen [8]. Legislation in most countries sets a maximum limit of 10 µg kg^−1^ for OTA in fresh and dried grapes. Among the potentially ochratoxin A-producing fungal species frequently found in grapes and raisins are *Aspergillus niger* and *Aspergillus carbonarius* [6]. Of these moulds, *A. carbonarius* is considered the main species responsible for the presence of OTA in dried grapes [9,10]. OTA accumulation takes place during the pre-harvest, harvest, and drying stages [11]. Synthetic fungicides, widely used to protect vineyards against many fungal diseases and prevent mycotoxin contamination, also raise additional concerns due to the risks they may pose to human health [12]. Similarly, traditional decontamination treatments involving sulphur dioxide (SO_2_) are limited due to the allergic reactions it can cause in sensitive consumers [13]. On the other hand, the quality of raisins can also be affected by the chemical pre-treatments frequently employed in the commercial production of raisins to accelerate the drying rate. These treatments consist of mixtures of alkaline solutions (such as sodium hydroxide, potassium carbonate, and sodium bicarbonate) and oil emulsions (such as vegetable oil and ethyl oleate) that alter the epicuticular wax layer of grapes, which promotes moisture loss [14]. The residues of these chemicals can remain in the final product, posing a potential health risk to consumers. There are also issues associated with handling large quantities of these corrosive substances [14]. To overcome these drawbacks, several studies have focused on investigating alternative techniques to reduce fungal contamination in grapes [15,16]. Nevertheless, research regarding novel pre-drying treatments aimed at enhancing the drying process remains limited [17].

UV-C light and ozone in gaseous and aqueous phases are among the decontamination agents that have been widely studied for fruit preservation [18,19]. These techniques have the advantage of leaving no chemical residues in the food matrix [20,21]. They can act directly on microorganisms by compromising their cellular components, including nucleic acids, proteins, and other biomolecules [18,22,23,24]. Both techniques can also activate defence systems in plant tissues, providing an additional indirect benefit by inhibiting the growth of phytopathogens [25,26]. Individual applications of ozone and UV-C light for reducing fungal spoilage in foods have been documented in several reports [27,28,29,30]. Nevertheless, only a few studies have evaluated the feasibility of using these techniques to inhibit or delay the growth of *A. carbonarius*, particularly with respect to controlling the fungus on grapes [31,32,33,34]. Previous studies on the Red Globe grape cultivar suggest that sequentially applying aqueous ozone followed by UV-C light at specific doses is a promising strategy for reducing the proliferation of *A*. *carbonarius* on this fruit [34]. In addition to their antimicrobial activity, ozone and UV-C light can alter the structure of the epidermis and mesocarp of grape tissues, depending on the dose applied [35,36,37]. Changes to the tissue structure could affect its transport properties, facilitating moisture removal. Therefore, these stress factors could also act in favour of the fruit drying. The use of these techniques for this purpose has received little attention [38,39]. Thus, this research aimed to evaluate the application of ozone and UV-C light treatments, either individually or in sequence, prior to drying Sultanina grapes, to reduce the incidence of *Aspergillus carbonarius* and accelerate the drying process. The impact of these pre-treatments on the structure of epicuticular waxes and colour of the dehydrated grapes was also assessed.

## 2. Materials and Methods

### 2.1. Raw Material Preparation and Characterisation

Fully ripened seedless grapes (*Vitis vinifera*) cv. Sultanina were supplied by a vineyard of the San Juan National Institute of Agriculture Technology (San Juan, Argentina). Fresh grapes were characterised in terms of soluble solids content, titratable acidity, and moisture content. The pH was determined using an Orion PerpHect^®^LogRTM model 310 pH meter (Thermo Fisher Scientific, Waltham, MA, USA), which was calibrated with pH 7.0 and 4.0 buffer solutions (Biopack, Zárate, Argentina). Grapes were ground, and pH measurements were directly taken from the fruit puree. Soluble solids content of grounded grapes was measured using a PR-101 digital refractometer (Atago, Tokyo, Japan), which had been calibrated with distilled water beforehand. Titratable acidity was determined using the potentiometric titration method. Briefly, 5 g of ground grapes were mixed with 50 mL of distilled water previously heated up to 80 °C. After 30 min, the mixture was filtered and titrated with a sodium hydroxide solution 0.1 N (Merck, Darmstadt, Germany) until reaching a pH value of 8.1 ± 0.2. Moisture content was determined by the gravimetric method, vacuum-drying the grapes at ~200 Torr and 60 ± 2 °C until constant weight [40,41]. All measurements were performed in triplicate. For the studies of drying kinetics, the equatorial and longitudinal diameters of individual grapes were also recorded using a gauge. The grapes were removed from the bunch and sorted by size, with any fruit showing skin damage and/or microbiological deterioration being discarded. The selected grapes were then used immediately for the different experiments.

### 2.2. Inoculation of Grapes with Aspergillus carbonarius

*Aspergillus carbonarius* RC131 was provided by the collection of the Department of Industry, Faculty of Exact and Natural Sciences, University of Buenos Aires. The fungal strain was cultivated on malt extract agar (Britania, Ciudad Autónoma de Buenos Aires, Argentina) and incubated for 7 days at 25 ± 1 °C. The conidia were collected from the culture medium by washing it with sterile distilled water. The resulting suspension was then filtered through sterile gauze to remove large mycelium fragments. The concentration of conidia in the suspension was quantified by microscopic observation using a Neubauer chamber (Exacta, Munich, Germany). The suspension was then diluted with sterile distilled water to achieve a final concentration of approximately 10^3^ conidia mL^−1^.

Fresh grapes were sanitised for 2 min with a sodium hypochlorite solution (200 mg L^−1^, Limsis, Villa Maipú, Argentina), rinsed 3 times with distilled water, and dried with filter paper. A small cross-shaped wound was then made in the equatorial zone of each grape using a sterile scalpel, into which 20 μL of the conidial suspension was inoculated. The inoculated grapes were placed inside a Class II Biosafety Hood (Tecnoequipar, Ciudad Autónoma de Buenos Aires, Argentina) for 2 h to facilitate adhesion of the fungus to the fruit surface. They were then packed into 26 × 19 × 6 cm trays (20 fruits per tray) and kept at 20 ± 1 °C for 24 h prior to undergoing the pre-treatments.

### 2.3. Pre-Treatments

To perform ozone pre-treatments, a corona discharge ozone generator (UTK-O-4/8, Unitek S.A., Mar del Plata, Argentina) was used and fed with pure oxygen (99.5%, Oxígeno Central, Ciudad Autónoma de Buenos Aires, Argentina; 0.62 bar) at a flow rate of 5.0 L min^−1^, and the nominal capacity of the generator was set at 99% to achieve an ozone concentration of 16.5 ± 0.8 mg O_3_ L^−1^ in the outlet stream [42]. The generated ozone was then injected into the base of a bubble column containing 1.25 L of distilled water at 20 ± 1 °C and 20 grapes (~150 g). The samples were exposed to ozone for 10 min. After a stabilisation period of ~5 min, the maximum concentration of dissolved ozone in the column was 4.1 ± 0.4 mg O_3_ L^−1^, as determined by the indigo spectrophotometric method [43]. The fruit was gently dried with absorbent paper after ozonisation.

UV-C light pre-treatments were carried out in a closed cabinet (height 0.4 m, length 1.0 m, width 0.3 m) equipped with four germicidal lamps (TUV-15W, G15 T8, Philips, Amsterdam, The Netherlands; maximum emission at 253.7 nm), one pair at the top and the second pair at the bottom. The cabinet inside was foiled with aluminium film and had a ventilation system (Ecoclima, Ciudad Autónoma de Buenos Aires, Argentina) to prevent the grapes from overheating [37]. The average temperature reached during pre-treatments was 27 ± 2 °C. For each experiment, 20 grapes were placed on a shelf located 8 cm away from the lamps, made of a transparent Teflon film (FEP 3D LD-002R-LD-002H, 95% UV light transmissivity, Creality, Shenzhen, China), allowing for simultaneous irradiation of the fruit from both sides. Samples were exposed for 30 min, corresponding to a fluence of 30.3 kJ m^−2^. The fluence was determined in triplicate, according to the methodology described by Rahn [44].

In the combined pre-treatment, sequential application of aqueous ozone (10 min-4.1 ± 0.4 mg O_3_ L^−1^) followed by exposure to UV-C light (30 min, 30.3 kJ m^−2^) was investigated.

The UV-C fluence and aqueous ozone concentration/exposure time assayed in the individual and sequential pre-treatments were chosen based on prior microbiological studies in Red Globe grapes [34], and a previous screening in which the effect of different doses on the drying rate was assessed.

To contrast the effect of the pre-treatments proposed in this work with the conventional drying pre-treatments, a chemical dipping was also evaluated. The grapes were immersed in a solution containing 2% (*v*/*v*) of ethyl-oleate (Deltaquim SRL, Ciudad Autónoma de Buenos Aires, Argentina) and 2.5% (*v*/*v*) of potassium carbonate (Merck, Darmstadt Germany) for 3 min at 40 ± 1 °C, identified as EtOl-K_2_CO_3_ pre-treatment.

### 2.4. Incidence of Aspergillus carbonarius in Artificially Contaminated Grapes During Storage

The development of *A. carbonarius* in inoculated untreated (control) and treated grapes was evaluated throughout 15 days at 20 ± 1 °C. For these storage studies, the fruit was packed in air-permeable polypropylene trays measuring 26 cm × 19 cm × 6 cm (with 20 fruits per tray). The growth of *Aspergillus carbonarius* on inoculated grapes, both pre-treated and non-treated, was monitored daily by visual inspection over the course of 15 days of storage, considering the presence or absence of fungal mycelium development beyond the severity of the infection. Three treatment replicates were evaluated, monitoring a total of sixty grapes for each condition tested. Results were reported as the percentage of infected grapes.

### 2.5. Drying Kinetics Studies

Untreated fruit (control) and grapes subjected to the different pre-treatments were immediately dried in a laboratory-scale convective dryer described previously by González-Fésler et al. [40]. Drying experiments were carried out in duplicate at an air temperature of 60 ± 1 °C, a relative humidity of 9.2 ± 1.3%, and a constant air velocity of 3 m s^−1^ to ensure conditions of internal resistance to mass transfer. The weights of the grapes were recorded on an analytical balance (Adventurer, Ohaus Corp., Parsippany, NJ, USA) every hour with a precision of ±0.0001 g until a moisture content of approximately 19–20% w.b. was reached (values of moisture content close to the maximum permissible for the commercialisation of raisins) [45]. Drying curves were plotted as the moisture ratio (M_R_) vs. time (min). M_R_ was calculated according to Equation (1):

(1) $$M_{R}=\frac{m_{t}-m_{e}}{m_{0}-m_{e}}$$

where m_t_ is the moisture content at a given time of drying, m_0_ the initial moisture content, and m_e_ the equilibrium moisture content with drying air (all expressed on a dry basis). It was assumed that the m_e_ was approximately 0 g/g dry mass [46,47].

The experimental drying curves (M_R_ vs. time) were fitted with the following semitheoretical and empirical models derived from Fick’s second law of diffusion or Newton’s law of cooling, which are frequently used to describe the drying kinetics of agricultural materials [48]:

Lewis model:

(2) $$M_{R}=e^{-kt}$$

Page model:

(3) $$M_{R}=e^{-kt^{n}}$$

Henderson and Pabis model:

(4) $$M_{R}=ae^{-kt}$$

where t is the drying time, and k, n, and a are the drying constants.

### 2.6. Ultrastructure Observations

Environmental scanning electron microscopy (ESEM) was used to assess the impact of the pre-treatments on the epicuticular waxes of the grape epidermis immediately after treatments, according to Fava et al. [36]. Pre-treated and non-treated whole grapes without prior preparation were placed in the sample holder of an environmental scanning electron microscope (Electroscan 2010, Wilmington, DE, USA) and observed at a pressure of 2.0–5.0 Torr and a voltage acceleration of 10.0–20.0 kV. Three samples were analysed for each condition tested.

### 2.7. Colour Measurements

The surface colour of the dried grapes, with and without pre-treatments, was determined using a handheld tristimulus reflectance spectrocolourimeter (Minolta Co., model CM-700d, Tokyo, Japan). Colour measurements were performed 24 h after drying, using a measuring aperture of 0.3 cm, D65 illuminant, an observer angle of 2°, and a black background. L* (lightness), a* (redness/greenness), and b* (yellowness/blueness) values were recorded. Chroma (C*) and hue angle (h*) functions were also calculated using the equations described by Gómez et al. [49]. Six dried grapes were analysed for each condition, taking four readings in different regions of each sample.

### 2.8. Statistical Analysis

The statistical analysis was performed using the Infostat version 2016 software (Universidad Nacional de Córdoba, Córdoba, Argentina). A two-way ANOVA was used to compare the effect of pre-treatment and storage time on fungal decay, and a Duncan’s test was performed to compare the means. A multivariate analysis of variance (MANOVA) followed by a post hoc Hotelling test with Bonferroni correction was used to evaluate the effect of pre-treatments on the colour of the raisins. Principal component analysis (PCA) was used to identify the relationship of colourimetric parameters/functions and samples. In all cases, the significance level was set at *p* < 0.05.

Nonlinear regressions were performed using Origin version 9.1 (OriginLab Corporation, Northampton, MA, USA) based on the Levenberg–Marquardt algorithm. The models’ goodness of fit was evaluated using the statistical parameters R^2^_adj_ (adjusted coefficient of determination) and RMSE (root mean square error).

## 3. Results and Discussion

### 3.1. Fruit Characterisation

Fresh grapes were characterised in terms of their soluble solids content (20.0 ± 0.2 °Brix), pH (3.5 ± 0.1), titratable acidity (512.3 ± 11.3 mg tartaric acid 100 g^−1^ wet matter), and moisture content (81.3 ± 0.8 g H_2_O 100 g^−1^ wet matter), which was consistent with the fully yellow stage for harvest, according to Khalil et al. [50].

### 3.2. Effect of Pre-Treatments on Aspergillus carbonarius Incidence

**Figure 1:**
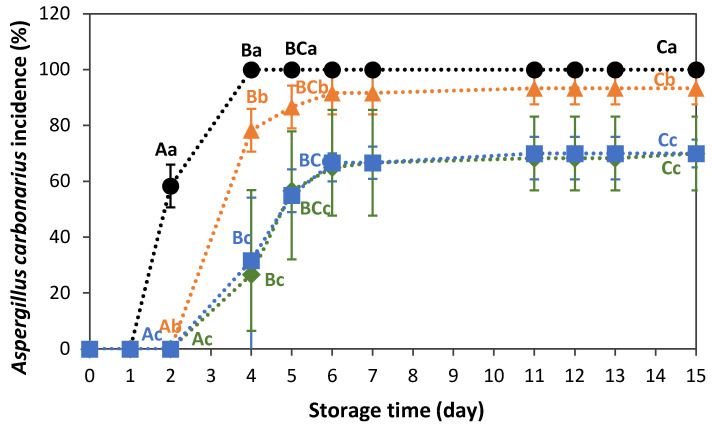
Incidence of *A. carbonarius* (10^3^ conidia mL^−1^) in Sultanina grapes during storage at 20 ± 1 °C of control (●), 10 min-4.1 mg O_3_ L^−1^ (▲), 30.3 kJ m^−2^ UV-C (■), and 10 min-4.1 mg O_3_ L^−1^ + 30.3 kJ m^−2^ UV-C (◆). Vertical bars represent the standard deviation of the means (n = 3, 20 fruits inspected per replicate). Uppercase letters indicate significant differences among storage day and lowercase letters indicate significant differences among treatments (*p* < 0.05).

Figure 1 shows the evolution of the incidence of *A. carbonarius* over storage in inoculated Sultanina grapes, both with and without pre-treatments. No significant interaction between “treatment” and “storage time” was observed (F_9,30_ = 0.5; *p* = 0.85), but the main effects of each factor were statistically significant (F_3,30_ = 66.1, *p* < 0.0001 for pre-treatment, and F_3,30_ = 4.1, *p* = 0.01 for storage time). Control grapes (inoculated without pre-treatments) showed a significant increase in fungal infection from day 2 of storage, with 100% of the fruit decaying by day 4. Grapes that were exposed to individual and sequential pre-treatments showed a one-day delay in the onset of infection, as well as significantly lower infection percentages throughout storage, compared to the control fruit. Of the pre-treatments evaluated, ozonisation with aqueous ozone (10 min-4.1 ± 0.4 mg O_3_ L^−1^) was the least effective strategy for controlling *A. carbonarius* growth, reaching a maximum percentage of infected fruit of 92% on day 6 of storage. From day 4 onwards, grapes pre-treated with 30.3 kJ m^−2^ UV-C or 10min-4.1 ± 0.4 mg O_3_ L^−1^ + 30.3 kJ m^−2^ UV-C showed a significantly lower mould incidence than ozonated samples. Additionally, no significant differences were found in the application of individual or combined UV-C light pre-treatments. The infection rate in these samples was approximately 30% lower than in the control group from day 6 until the end of storage.

*Aspergillus carbonarius* contains melanins, which are dark-brown pigments produced by the oxidative polymerisation of phenolic and indolic monomers. These pigments have been shown to play a role in the invasion of host tissue [51]. It has also been demonstrated that these pigments provide increased protection against various environmental stress factors, including solar radiation, UV light, oxidising agents, and heavy metals [27,52,53,54]. Ozone in the aqueous and gas phase has been found to cause a loss of dark-brown pigmentation in conidia of *A. carbonarius* and other fungal species [34,55], which could be related to chemical changes in melanins. Despite this effect, previous in vitro studies have shown that *A. carbonarius* conidia are more sensitive to UV-C light than aqueous ozone [34]. In line with this, our current study also showed that UV-C light is more effective than aqueous ozone at controlling *A. carbonarius* development in Sultanina grapes during storage. We observed the same pattern of effectiveness in our previous studies, in which we assessed these treatments individually in Red Globe grapes [34]. In addition, Akbar et. al. [56] reported a minimal effect of gaseous ozone treatments (400 and 600 mg O_3_ L^−1^, 60 min) on *A. carbonarius* growth in artificially contaminated green coffee beans with different water activities (0.75, 0.9, and 0.95) during storage at 30 °C, which is consistent with our results. Meanwhile, Valero et al. [27] observed in in vitro studies that *A. carbonarius* showed a lower sensitivity to UV-C light than other species isolated from grapes, including *Aspergillus niger*. Although both species of *Aspergillus* section Nigri contain melanin, the greater UV-C-resistance of *A. carbonarius* spores relative to *A. niger* was linked to its thicker cell walls [27]. On the other hand, in grapes cv. Napoleon, it was found that even a lower dose of UV-C light than that applied in our work (6 kJ m^−2^) led to a significant reduction in the development of *A. carbonarius* and *Aspergillus tubingensis* during storage at 22 °C. The inhibitory effect of UV-C light on the mould’s growth was partly associated with the induction of phytochemical compounds with antimicrobial properties, such as *trans*-resveratrol, in response to the antifungal factors [32].

The selected sequential treatment of aqueous ozone and UV-C light did not provide an additional antifungal effect on *A. carbonarius* in Sultanina grapes compared to the individual application of UV-C light. This outcome contrasts with our previous observations in Red Globe grapes [34]. The evaluation of the effect of different inactivation agents on microorganisms attached to a fruit matrix is complex, since the fungal inhibition is also dependent on the fruit’s physiological response. Several factors are involved in the fruit’s response to fungal attack, such as fruit cultivar, pre-harvest conditions, and the treatment dose applied, among others [25,26,57]. The same treatment and storage conditions used in our previous study [34] led to different responses in fungal inhibition when a different grape cultivar was evaluated. Therefore, the grape cultivar could influence the fungus’s response to the treatments, as was observed by other authors when evaluating different postharvest decontamination techniques [58,59]. Deeper studies on the physiological mechanisms triggered by the treatments on different cultivars should be performed to provide a deeper understanding of these differences.

### 3.3. Ultrastructure Observations of Epicuticular Waxes

**Figure 2:**
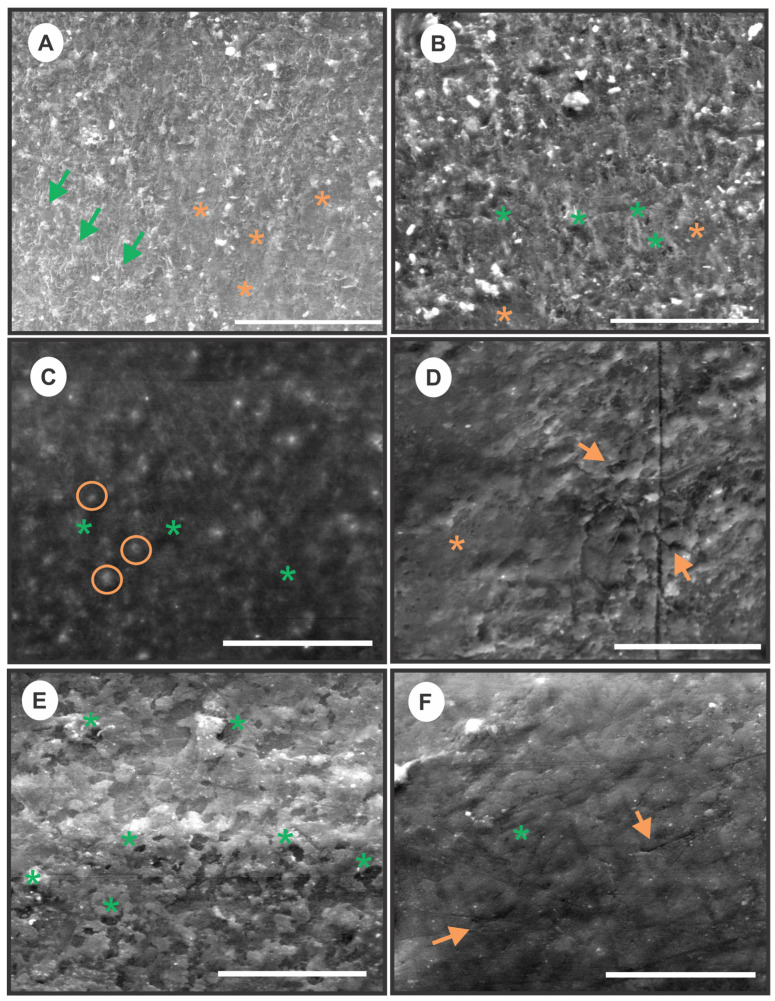
ESEM micrographs of grapes tissues of control (**A**,**B**) and pre-treated with 30.3 kJ m^−2^ UV-C (**C**), 10 min-4.1 mg O_3_ L^−1^ (**D**), 10 min-4.1 mg O_3_ L^−1^ + 30.3 kJ m^−2^ UV-C (**E**), and EtOl-K_2_CO_3_ (**F**). Green arrows: platelets; orange asterisks: smooth areas; green asterisks: pores; orange circles: excrescencies; orange arrows: cracks. Scale (**A**–**F**): 50 μm.

Figure 2 shows ESEM images of the pre-treated and untreated grape surfaces. Micrographs of untreated samples revealed a dense and heterogeneous layer of epicuticular waxes, with irregular, three-dimensional morphologies that covered the cuticle (Figure 2A,B). Mostly, the waxy network consists of overlapping crystalloid platelets (green arrows), usually interconnected, with air spaces between them, which emerge from the berry surface at an acute angle without a specific orientation. These images match the description of other authors, who, in addition to this, have reported distinctive morphological features depending on the grape cultivar [36,60,61,62]. Also, the micrographs show smoother regions (orange asterisks) that are difficult to distinguish using ESEM but could represent amorphous films, soft waxes, and/or wax-free surfaces, indicating the great micromorphological diversity of the waxes [63]. In this respect, Rogiers et al. [64] also observed areas with amorphous waxes on the top of the crystalline platelets of varying extent (10 to 80% of the total grape surface) in other grape varieties. However, these authors related the presence of amorphous waxes to regions of the grape surface exposed to the sun or areas where the berries were in contact with other berries.

The topography of 30.3 kJ m^−2^ UV-C treated grapes showed marked differences in the distribution of waxes when compared to untreated ones, mainly characterised by the presence of smoother areas (mainly attributed to wax-free regions and pores) intermixed with small clusters of waxes (including platelets) or excrescences (orange circles) of varied size and randomly distributed (Figure 2C). Similar results on the effect of UV-C light in epicuticular waxes of grapes cv. Isabella were reported in a previous work [36]. Exposure to 10 min-4.1 mg O_3_ L^−1^ treatment caused a redistribution of epicuticular waxes and a modification in the wax morphology (Figure 2D). The waxes appeared to be pressed against the entire surface of the cuticle, forming a rather smoother film (orange asterisks). Some small pores and incipient ruptures in the film were also visible. The change in the morphology of the waxes could be attributed to changes in their chemical composition [63]. The literature reported a partial removal of epicuticular waxes in O’Neal blueberries subjected to aqueous ozone treatments, as well as chemical modification of the waxes [37,65]. The surfaces of grapes exposed to 10 min-4.1 mg O_3_ L^−1^ + 30.3 kJ m^−2^ UV-C were observed with a different topography than the individual pre-treatments. The epicuticular wax layer was arranged in numerous fairly loose or isolated aggregates surrounded by small pores, suggesting a greater degree of alteration than that caused by the individual pre-treatments (Figure 2E). Dipping grapes in EtOl-K_2_CO_3_ caused a marked alteration in the continuity of the wax layer. As a consequence of wax solubilisation by ethyl-oleate, several channels would be formed within the wax network, resulting in a striated surface pattern, where the waxes were surrounded by numerous interconnected channels or pores. Some cracks on the surface were also observed (orange arrows) (Figure 2F). Similarly, Possingham [60] found a reorientation of wax platelets parallel to the berry surface and a reduction in the effective thickness of the wax layer in cv. Sultanina grapes dipped in ethyl-oleate solutions.

### 3.4. Effect of Pre-Treatments on Drying Kinetics

**Figure 3:**
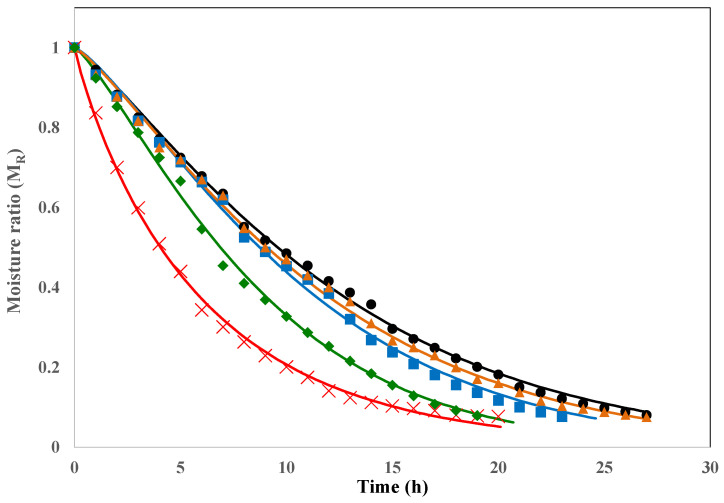
Drying curves of Sultanina grapes subjected to different pre-treatments. The symbols correspond to the experimental data and the solid lines to fitted values derived from Page model. Control (●, ▬), 10 min-4.1 mg O_3_ L^−1^ (▲, ▬), 30.3 kJ m^−2^ UV-C (■, ▬), 10 min-4.1 mg O_3_ L^−1^ + 30.3 kJ m^−2^ UV-C (◆, ▬), and EtOl-K_2_CO_3_ (×, ▬).

Drying curves (moisture ratio, M_R_, vs. time) for the control and pre-treated grapes are shown in Figure 3. In all cases, the drying process took place in the falling drying rate period, as has been previously reported for grapes and other fruits dried in similar conditions [40,46]. The pre-treatments evaluated increased the drying rate of the grapes when compared to the control (untreated grapes), except for samples pre-treated with aqueous ozone (10 min-4.1 mg O_3_ L^−1^).

For comparative purposes, the drying curves were modelled with the Lewis, Page, and Henderson and Pabis models, which have been previously used to describe the drying kinetics of grapes [46,66]. The constants estimated from the modelling and the statistical parameters used to assess the adjustment and performance of the models are presented in Table 1. All drying curves were successfully fitted with the models evaluated, as evidenced by the high values of R^2^_adj_ (>0.97) and low RMSE values (<0.048) obtained. The best fit was achieved by applying the Page model, in agreement with the findings of other authors when studying grape-drying kinetics [66,67]. The higher dehydration rate observed in grapes pre-treated with 10 min-4.1 mg O_3_ L^−1^ + 30.3 kJ m^−2^ UV-C or EtOl-K_2_CO_3_ was mainly related to an increase in the k parameter value obtained from this model. Based on the Page parameters and the final M_R_ (19–20% w. b.) for each sample group, the theoretical drying time reductions compared with the untreated samples were ~13% and ~31% for grapes pre-treated with individual 30.3 kJ m^−2^ UV-C and the combined 10 min-4.1 mg O_3_ L^−1^ + 30.3 kJ m^−2^ UV-C treatments, respectively, and ~39% for grapes exposed to the conventional pre-treatment with EtOl-K_2_CO_3_. To our knowledge, the combined application of these non-thermal techniques prior to food drying has not been investigated before. However, the positive impact of UV-C light in accelerating drying has been reported for other food matrices.

For instance, Chandrakar and Banerjee [39] found that pre-treatments with UV-C light at 0.23 J cm^−2^ on *Costus pictus* D. leaves resulted in a reduction in drying time of approximately 60 and 25% when the leaves were subsequently dehydrated in a hot air dryer at 40 and 60 °C, respectively. Forouzanfar et al. [38] observed a depletion in drying time of approximately 37 to 50% in mushrooms (*Agaricus bisporus*) dried at 50 °C and previously exposed to UV-B light for 30 to 90 min (fluence not reported), respectively.

The main barrier to the transfer of moisture from the internal tissues of grapes to the outside is found in the epidermis [61]. The grape epicarp consists of one stratum of epidermal cells and three to six layers of subepidermal cells [36]. The layer of epicuticular waxes (composed of oleanolic acid, fatty acids, paraffins, aldehydes, alcohols, and esters) covering the epidermal cells acts as a hydrophobic barrier to water removal, slowing the dehydration process [68]. The ultrastructure study allowed for identifying the different damages occasioned to the epicuticular wax layer by the pre-treatments (Figure 2). Although all pre-treatments caused alterations in the epicuticular waxes, the wax aggregates and formation of numerous pores in the 10 min-4.1 mg O_3_ L^−1^ + 30.3 kJ m^−2^ UV-C treated grapes and the interconnected channels and cracks observed in the EtOl-K_2_CO_3_ treated grapes would decrease the resistance to mass transfer through the epidermis. This fact could at least partially explain the faster drying rate observed in these samples compared to the control and those exposed to individual pre-treatments. These results are also consistent with some findings in the literature. The partial removal or redistribution of epicuticular waxes and the generation of microcracks facilitate the dehydration of grapes, but also, changes in the chemical composition or hydrophobicity of the waxes contribute to the moisture loss during drying [60,69]. Possingham [60] hypothesised that the reorientation of wax platelets parallel to the surface in EtOl-K_2_CO_3_-treated grapes could facilitate the contact of hydrophilic groups, thereby enhancing water removal. In the case of 10 min-4.1 mg O_3_ L^−1^-treated grapes, the wax film covering the entire surface of the cuticle continues to act as a barrier to moisture passage without impacting the drying rate compared to the control. Pre-treatment with 30.3 kJ m^−2^ UV-C alone slightly reduced the rate of moisture removal. It should be noted that the effectiveness of pre-treatments to accelerate the drying process could be cultivar-dependent. In previous studies of our group, differences in the drying rates have been found among grape cultivars when pre-treated with 30.3 kJ m^−2^ of UV-C light [70]. These differences in the dehydration rates across cultivars could be partly attributed to variations in the structural characteristics of the wax layer and epidermal and subepidermal cells that can be found among grape genotypes [61,71].

### 3.5. Colour of Pre-Treated Raisins

**Figure 4:**
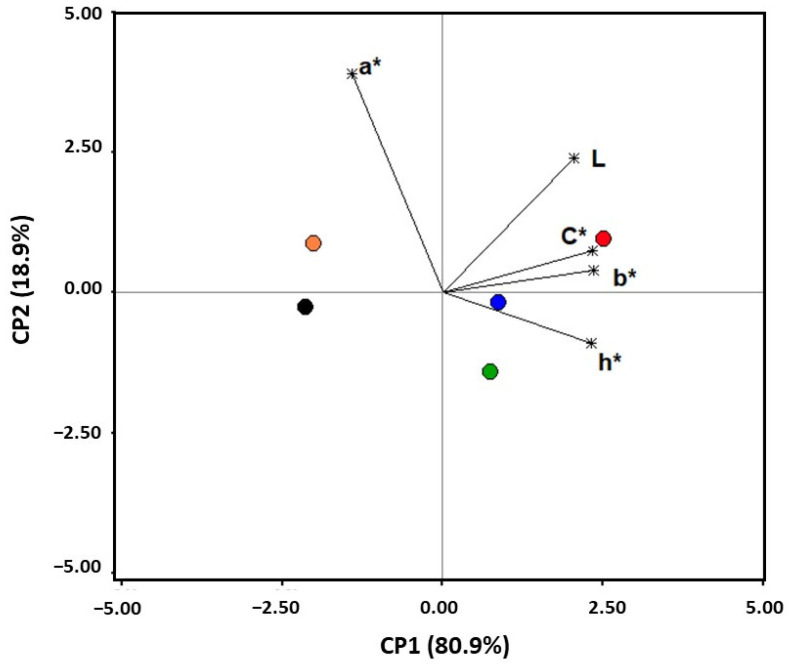
Principal component analysis (PCA) bi-plot of the CIELAB coordinates (L, a*, and b*) and colour functions (C* and h*) of dehydrated Sultanina grapes subjected to different pre-treatments. Control (●) and pre-treated raisins with 30.3 kJ m^−2^ UV-C (●), 10 min-4.1 mg O_3_ L^−1^ (●), 10 min-4.1 mg O_3_ L^−1^ + 30.3 kJ m^−2^ UV-C (●), and EtOl-K_2_CO_3_ (●).

The CIELab colourimetric parameters and functions of Sultanina grapes that were exposed to the different pre-treatments and subsequently dehydrated are shown in Table 2. Significant differences in colour between the control and pre-treated raisins were observed, except for grapes exposed to ozone (F_5,22_ = 55.8, *p* < 0.0001). In the Principal Component Analysis (Figure 4), the first component (PC1) explained 80.9% of the total variance, classifying the samples primarily according to their b*, h, and C values and, to a lesser extent, their L* values. Thus, the variation in the colour of pre-treated raisins exposed to 30.3 kJ m^−2^ UV-C, 10 min-4.1 mg O_3_ L^−1^ + 30.3 kJ m^−2^ UV-C, or EtOl-K_2_CO_3_ was mainly associated with slight increases in the b*, h, and C values compared to the control. Changes in the colourimetric parameters and functions indicated that the samples exposed to the aforementioned pre-treatments were less brown than the non-pre-treated raisins.

Grapes are prone to develop browning during convective drying due to enzymatic and non-enzymatic browning reactions [72]. Polyphenoloxidase (PPO) activity has been identified as a key enzyme involved in the development of enzymatic browning on the surfaces of grapes. Residual PPO activity has been found in Sultanina grapes heated at 60 °C [73,74], thus showing that enzymatic browning may have occurred during the drying process. Furthermore, the rate of enzymatic and non-enzymatic browning increases with an increasing water activity (a_w_) over the range of 0.3 and 0.8. At higher a_w_ values, enzymatic browning reactions are favoured over non-enzymatic ones [72].

According to our results, the pre-treatment with 30.3 kJ m^−2^ UV-C and 10 min-4.1 mg O_3_ L^−1^ + 30.3 kJ m^−2^ UV-C light helped in slowing down the browning of raisins, in a similar way to the conventional chemical pre-treatment with EtOl-K_2_CO_3_. Previous studies have also reported a depletion of browning in raisins treated with EtOl-K_2_CO_3_ solutions [75,76]. It has been suggested that the EtOl-K_2_CO_3_ solution increases the pH in the grape surface, which could reduce the non-enzymatic reactions [77]. In addition, it has also been hypothesised that the increase in drying rate by the chemical pre-treatment led to a rapid concentration of sugars in the grapes, which would exert an inhibitory effect on PPO activity, thereby limiting the enzymatic browning [75]. Similarly, it is likely that the reduction in browning from the 10 min-4.1 mg O_3_ L^−1^ + 30.3 kJ m^−2^ UV-C pre-treatment could also be linked to the decrease in drying time. Moreover, some reports indicate that both ozone and UV-C light can cause a direct inhibition of PPO enzymes [78,79].

## 4. Conclusions

The pre-treatment with 30.3 kJ m^−2^ of UV-C light was more effective than the application of aqueous ozone (10 min-4.1 mg O_3_ L^−1^) in inhibiting the growth of *A. carbonarius* on Sultanina grapes throughout storage at 20 °C. Applying these treatments in sequential combination did not provide an additional significant benefit on the fungus incidence. However, grapes subjected to the combined pre-treatment exhibited higher dehydration rates than those pre-treated with ozone and UV-C light alone, with the reduction in drying time slightly lower than that achieved with the traditional chemical pre-treatment of EtOl-K_2_CO_3_. This enhancement in drying rate was partly linked with marked alterations in the grape’s wax layer. Moreover, the combined pre-treatment helped in slowing down the browning during drying. These findings suggest that the use of aqueous ozone and UV-C light in combination could be an environmentally friendly alternative for raisin production, for both improving the microbiological quality of grapes and accelerating the drying process.

## Figures and Tables

**Table 1:** Parameters of Lewis, Page, and Henderson and Pabis models and statistical parameters obtained from fitting the drying curves of grapes subjected to different pre-treatments.

Sample	Lewis			Page				Henderson and Pabis			
	k	R^2^_adj_	RMSE	n	k	R^2^_adj_	RMSE	a	k	R^2^_adj_	RMSE
Control	0.078 ± 0.002	0.9857	0.0344	1.21 ± 0.02	0.045 ± 0.002	0.9908	0.0126	1.06 ± 0.01	0.083 ± 0.002	0.9900	0.0287
30.3 kJ m^−2^ UV-C	0.087 ± 0.002	0.9737	0.0481	1.29 ± 0.03	0.042 ± 0.003	0.9956	0.0184	1.07 ± 0.02	0.094 ± 0.003	0.9803	0.0416
10 min-4.1 mg O_3_ L^−1^	0.082 ± 0.002	0.9845	0.0369	1.23 ± 0.02	0.046 ± 0.002	0.9978	0.0111	1.06 ± 0.02	0.087 ± 0.002	0.9886	0.0310
10 min-4.1 mg O_3_ L^−1^ + 30.3 kJ m^−2^ UV-C	0.110 ± 0.003	0.9794	0.0427	1.26 ± 0.03	0.061 ± 0.003	0.9973	0.0121	1.06 ± 0.02	0.118 ± 0.004	0.9860	0.0354
EtOl-K_2_CO_3_	0.163 ± 0.003	0.9949	0.0195	0.91 ± 0.02	0.194 ± 0.007	0.9975	0.0118	0.98 ± 0.01	0.159 ± 0.003	0.9953	0.0186

Mean values ± standard deviation. Lewis model: k = [h^−1^]. Page model: k = [h^−n^], n [dimensionless]. Henderson and Pabis: k = [h^−1^], a [dimensionless].

**Table 2:** Average values of colourimetric parameters and functions of dehydrated Sultanina grapes subjected to different pre-treatments.

Sample	L*	a*	b*	C*	h*	
Control	33.3 ± 1.2	6.9 ± 1.0	11.8 ± 1.8	13.6 ± 2.0	59.6 ± 0.8	A
30.3 kJ m^−2^ UV-C	34.1 ± 2.1	7.1 ± 1.7	15.9 ± 2.4	17.4 ± 2.9	66.2 ± 2.4	B
10 min-4.1 mg O_3_ L^−1^	33.8 ± 0.9	7.2 ± 0.8	12.1 ± 0.9	14.1 ± 1.1	59.2 ± 2.2	A
10 min-4.1 mg O_3_ L^−1^ + 30.3 kJ m^−2^ UV-C	33.9 ± 2.3	6.2 ± 0.4	14.9 ± 1.2	16.2 ± 1.1	67.3 ± 1.7	B
EtOl-K_2_CO_3_	35.5 ± 1.7	6.7 ± 0.9	17.6 ± 1.2	18.9 ± 1.4	69.1 ± 1.7	B

Mean values ± standard deviation. Different letters indicate significantly different optical properties (*p* < 0.05).

## Data Availability

The original contributions presented in this study are included in the article. Further inquiries can be directed to the corresponding authors.
